# Supplementary material for: Pyruvate Dehydrogenase Kinase 1 inhibition mediated oxidative phosphorylation enhancement in cartilage promotes osteoarthritis progression
Source: BMC Musculoskelet Disord. 2023 Jul 20;24:597. doi: 10.1186/s12891-023-06585-6 (PMC10357736; doi:10.1186/s12891-023-06585-6)
Supplement: Supplementary file 1 — Additional file 1. [file 12891_2023_6585_MOESM1_ESM.zip › BP.Gsea.1653623667859/GOBP_MAINTENANCE_OF_BLOOD_BRAIN_BARRIER.html]

Details for gene set GOBP\_MAINTENANCE\_OF\_BLOOD\_BRAIN\_BARRIER[GSEA]

|  || Dataset | 55457\_collapsed\_to\_symbols.cls.cls #OA\_versus\_normal.cls.cls #OA\_versus\_normal\_repos |
| Phenotype | cls.cls#OA\_versus\_normal\_repos |
| Upregulated in class | normal |
| GeneSet | GOBP\_MAINTENANCE\_OF\_BLOOD\_BRAIN\_BARRIER |
| Enrichment Score (ES) | -0.5433972 |
| Normalized Enrichment Score (NES) | -1.8721621 |
| Nominal p-value | 0.0016949152 |
| FDR q-value | 0.06406768 |
| FWER p-Value | 0.824 |
Table: GSEA Results Summary

  

Fig 1: Enrichment plot: GOBP\_MAINTENANCE\_OF\_BLOOD\_BRAIN\_BARRIER      
 Profile of the Running ES Score & Positions of GeneSet Members on the Rank Ordered List

  

| SYMBOL | TITLE | RANK IN GENE LIST | RANK METRIC SCORE | RUNNING ES | CORE ENRICHMENT || 1 | ANGPT1 | angiopoietin 1 [Source:HGNC Symbol;Acc:HGNC:484] | 1467 | 0.433 | -0.0746 | No |
| 2 | ABCC8 | ATP binding cassette subfamily C member 8 [Source:HGNC Symbol;Acc:HGNC:59] | 2844 | 0.266 | -0.1574 | No |
| 3 | LSR | lipolysis stimulated lipoprotein receptor [Source:HGNC Symbol;Acc:HGNC:29572] | 3059 | 0.244 | -0.1519 | No |
| 4 | CLDN1 | claudin 1 [Source:HGNC Symbol;Acc:HGNC:2032] | 3089 | 0.241 | -0.1323 | No |
| 5 | JAM2 | junctional adhesion molecule 2 [Source:HGNC Symbol;Acc:HGNC:14686] | 5602 | 0.024 | -0.3253 | No |
| 6 | DMD | dystrophin [Source:HGNC Symbol;Acc:HGNC:2928] | 5605 | 0.023 | -0.3233 | No |
| 7 | SH3GL2 | "SH3 domain containing GRB2 like 2, endophilin A1 [Source:HGNC Symbol;Acc:HGNC:10831]" | 6007 | -0.005 | -0.3541 | No |
| 8 | LAMA2 | laminin subunit alpha 2 [Source:HGNC Symbol;Acc:HGNC:6482] | 7392 | -0.110 | -0.4516 | No |
| 9 | VCL | vinculin [Source:HGNC Symbol;Acc:HGNC:12665] | 7687 | -0.133 | -0.4623 | No |
| 10 | MBP | myelin basic protein [Source:HGNC Symbol;Acc:HGNC:6925] | 7819 | -0.143 | -0.4596 | No |
| 11 | SLC1A1 | solute carrier family 1 member 1 [Source:HGNC Symbol;Acc:HGNC:10939] | 8157 | -0.167 | -0.4706 | No |
| 12 | TJP1 | tight junction protein 1 [Source:HGNC Symbol;Acc:HGNC:11827] | 8523 | -0.195 | -0.4812 | No |
| 13 | GJA1 | gap junction protein alpha 1 [Source:HGNC Symbol;Acc:HGNC:4274] | 9325 | -0.258 | -0.5200 | Yes |
| 14 | JAM3 | junctional adhesion molecule 3 [Source:HGNC Symbol;Acc:HGNC:15532] | 9376 | -0.262 | -0.5001 | Yes |
| 15 | F11R | F11 receptor [Source:HGNC Symbol;Acc:HGNC:14685] | 9765 | -0.297 | -0.5033 | Yes |
| 16 | TJP2 | tight junction protein 2 [Source:HGNC Symbol;Acc:HGNC:11828] | 9772 | -0.298 | -0.4767 | Yes |
| 17 | CLDN5 | claudin 5 [Source:HGNC Symbol;Acc:HGNC:2047] | 9816 | -0.302 | -0.4526 | Yes |
| 18 | CDH5 | cadherin 5 [Source:HGNC Symbol;Acc:HGNC:1764] | 9946 | -0.316 | -0.4339 | Yes |
| 19 | TJP3 | tight junction protein 3 [Source:HGNC Symbol;Acc:HGNC:11829] | 10260 | -0.347 | -0.4268 | Yes |
| 20 | PECAM1 | platelet and endothelial cell adhesion molecule 1 [Source:HGNC Symbol;Acc:HGNC:8823] | 10304 | -0.351 | -0.3982 | Yes |
| 21 | LAMC1 | laminin subunit gamma 1 [Source:HGNC Symbol;Acc:HGNC:6492] | 10642 | -0.387 | -0.3893 | Yes |
| 22 | SLC12A2 | solute carrier family 12 member 2 [Source:HGNC Symbol;Acc:HGNC:10911] | 11744 | -0.540 | -0.4259 | Yes |
| 23 | IL6 | interleukin 6 [Source:HGNC Symbol;Acc:HGNC:6018] | 12460 | -0.730 | -0.4151 | Yes |
| 24 | ITGB1 | integrin subunit beta 1 [Source:HGNC Symbol;Acc:HGNC:6153] | 12566 | -0.780 | -0.3525 | Yes |
| 25 | ACTG1 | actin gamma 1 [Source:HGNC Symbol;Acc:HGNC:144] | 12613 | -0.806 | -0.2830 | Yes |
| 26 | CLDN3 | claudin 3 [Source:HGNC Symbol;Acc:HGNC:2045] | 12615 | -0.807 | -0.2098 | Yes |
| 27 | VEGFA | vascular endothelial growth factor A [Source:HGNC Symbol;Acc:HGNC:12680] | 12629 | -0.813 | -0.1370 | Yes |
| 28 | PTGS2 | prostaglandin-endoperoxide synthase 2 [Source:HGNC Symbol;Acc:HGNC:9605] | 12641 | -0.820 | -0.0635 | Yes |
| 29 | ACTB | actin beta [Source:HGNC Symbol;Acc:HGNC:132] | 12757 | -0.920 | 0.0111 | Yes |
Table: GSEA details [plain text format]

  

Fig 2: GOBP\_MAINTENANCE\_OF\_BLOOD\_BRAIN\_BARRIER      
 Blue-Pink O' Gram in the Space of the Analyzed GeneSet

  

Fig 3: GOBP\_MAINTENANCE\_OF\_BLOOD\_BRAIN\_BARRIER: Random ES distribution      
 Gene set null distribution of ES for **GOBP\_MAINTENANCE\_OF\_BLOOD\_BRAIN\_BARRIER**
